# α-Particle-induced DNA damage tracks in peripheral blood mononuclear cells of [^223^Ra]RaCl_2_-treated prostate cancer patients

**DOI:** 10.1007/s00259-020-05170-6

**Published:** 2021-02-04

**Authors:** S. Schumann, U. Eberlein, C. Lapa, J. Müller, S. Serfling, M. Lassmann, H. Scherthan

**Affiliations:** 1 Department of Nuclear Medicine, University of Würzburg, Würzburg, Germany; 2 Nuclear Medicine, Medical Faculty, University of Augsburg, Augsburg, Germany; 3 Bundeswehr Institute of Radiobiology affiliated to the University of Ulm, Munich, Germany

**Keywords:** DNA damage, Nuclear medicine, Dosimetry, α-Emitter, Biokinetics, Prostate cancer, [^223^Ra]RaCl_2_, γ-H2AX, 53BP1

## Abstract

**Purpose:**

One therapy option for prostate cancer patients with bone metastases is the use of [^223^Ra]RaCl_2_. The α-emitter ^223^Ra creates DNA damage tracks along α-particle trajectories (α-tracks) in exposed cells that can be revealed by immunofluorescent staining of γ-H2AX+53BP1 DNA double-strand break markers. We investigated the time- and absorbed dose-dependency of the number of α-tracks in peripheral blood mononuclear cells (PBMCs) of patients undergoing their first therapy with [^223^Ra]RaCl_2_.

**Methods:**

Multiple blood samples from nine prostate cancer patients were collected before and after administration of [^223^Ra]RaCl_2_, up to 4 weeks after treatment. γ-H2AX- and 53BP1-positive α-tracks were microscopically quantified in isolated and immuno-stained PBMCs.

**Results:**

The absorbed doses to the blood were less than 6 mGy up to 4 h after administration and maximally 16 mGy in total. Up to 4 h after administration, the α-track frequency was significantly increased relative to baseline and correlated with the absorbed dose to the blood in the dose range < 3 mGy. In most of the late samples (24 h – 4 weeks after administration), the α-track frequency remained elevated.

**Conclusion:**

The γ-H2AX+53BP1 assay is a potent method for detection of α-particle-induced DNA damages during treatment with or after accidental incorporation of radionuclides even at low absorbed doses. It may serve as a biomarker discriminating α- from β-emitters based on damage geometry.

**Supplementary Information:**

The online version contains supplementary material available at 10.1007/s00259-020-05170-6.

## Introduction

According to the data of the International Agency for Research on Cancer, prostate cancer is the fourth most common cancer world-wide and has been diagnosed in 1.3 million men in 2018, and accounts for 3.8% of all cancer deaths [1]. The majority of patients dying of prostate cancer display bone metastases and varying symptoms such as pain, pathological fractures, neurological disorders, spinal cord compression, and bone marrow failure, all of which are significantly impairing their quality of life [2, 3].

In 2013, the ALSYMPCA trial disclosed that [^223^Ra]RaCl_2_ treatment prolonged lives of castration-resistant prostate cancer patients with widespread bone metastatic disease. This led to marketing authorization for [^223^Ra]RaCl_2_ (Xofigo®) as the first α-emitting radiopharmaceutical [4, 5]. For the treatment, 55 kBq of [^223^Ra]RaCl_2_ per kilogram bodyweight is administered systemically by intravenous injection in up to six cycles.

As α-particles have a short range of less than 0.1 mm in soft tissue and a high linear energy transfer (LET), they have been found to induce complex chromosome aberrations [6, 7] and DNA damage [8, 9]. Administration of [^223^Ra]RaCl_2_ leads to ionizing radiation exposure of the targeted bone metastases as well as the blood and other organs and tissues [10]. Even a few hours and days after [^223^Ra]RaCl_2_ administration, there is activity remaining in the blood, leading to prolonged internal irradiation [11]. As hematotoxicity represents one of the known side effects after administration of [^223^Ra]RaCl_2_ [12], it is of great interest to characterize the DNA damage elicited by ^223^Ra and its progeny in patients’ peripheral blood mononuclear cells (PBMCs) for obtaining information on potential long-term side effects by persisting DNA damage.

The γ-H2AX+53BP1 focus assay has been proven useful to reliably quantify DNA double-strand break (DSB) damage, especially after low-dose irradiation. In this assay, cells are immunofluorescently stained for γ-H2AX and 53BP1 proteins that demarcate chromatin regions containing DSBs so that microscopically colocalizing foci of γ-H2AX and 53BP1 can be quantified and used as DSB biomarkers, e.g., after internal irradiation with β- and γ-emitting radionuclides [13–18]. After irradiation with α-emitting radionuclides, not only distinct foci but also γ-H2AX- and 53BP1-containing DNA damage tracks, so-called α-tracks, are detected in hit cells’ nuclei and likely reflect the DNA damage along the particle trajectory through the cell [8, 9, 19–21]. In a previous study, we observed that there is a linear relationship between the number of these α-tracks in PBMCs and the absorbed dose to the blood after internal ex vivo irradiation of blood samples with the α-emitter ^223^Ra [8]. A further study with the radium isotope ^224^Ra revealed that there is the same DNA damage induction for the two radium isotopes at a comparable absorbed dose to the blood [9]. Hence, it can be concluded that the number of α-tracks is a good measure for biological dosimetry after α-particle exposure. These ex vivo studies show that DNA damage geometry (small foci versus α-tracks) in PBMCs may help to reveal radionuclide incorporation in radiation and, e.g. nuclear power plants accidents thus serving as a LET-responsive biomarker. However, so far no in vivo data are available. Therefore, in the present study, we investigated the time- and absorbed dose-dependent in vivo DNA damage in PBMCs of prostate cancer patients during therapy with [^223^Ra]RaCl_2_.

## Methods

### Patients and blood sampling

Nine patients with metastatic castration-resistant prostate cancer receiving their first therapy cycle with [^223^Ra]RaCl_2_ (Xofigo®; Bayer, Leverkusen, Germany) were included in this prospective study. Patients were included if they had no history of disease of the hematopoietic system and/or did not receive radiation treatment or diagnostics with ionizing radiation up to 5 days before the 1st treatment. Each patient received a nominal activity of 55 kBq [^223^Ra]RaCl_2_ per kilogram body weight. Li-heparin blood collecting tubes (S-Monovette®; Sarstedt, Nümbrecht, Germany) were used for the blood collection.

One blood sample of each patient was taken as a baseline sample before [^223^Ra]RaCl_2_ was administered, in order to assess whether α-tracks were already present before irradiation. After injection, nominal blood sampling time points were 1.5 h, 3 h, 4 h, 24 h, 48 h (or 96 h), and approx. 4 weeks after administration. Due to individual patient management and variable treatment schedules, it was necessary to adjust the blood sampling time points in some cases. For example, the nominal time point at 48 h after administration had to be changed to 96 h after administration when the 48 h time point could not be met.

### Decay properties of ^223^Ra

The α-emitter ^223^Ra (half-life: 11.43 days) decays in six steps into the stable product ^207^Pb. Four of the radionuclides in the decay chain (^219^Rn, ^215^Po, ^211^Bi, ^211^Po) are also α-emitters and in total, four α-particles are emitted per decay [8]. All radionuclides in the decay chain are short-lived with half-lives of maximally 0.6 h (^211^Pb). More detailed information on half-lives and energy deposition per transition for ^223^Ra and its progeny is provided in a previously published ex vivo study [8].

**Fig. 1:**
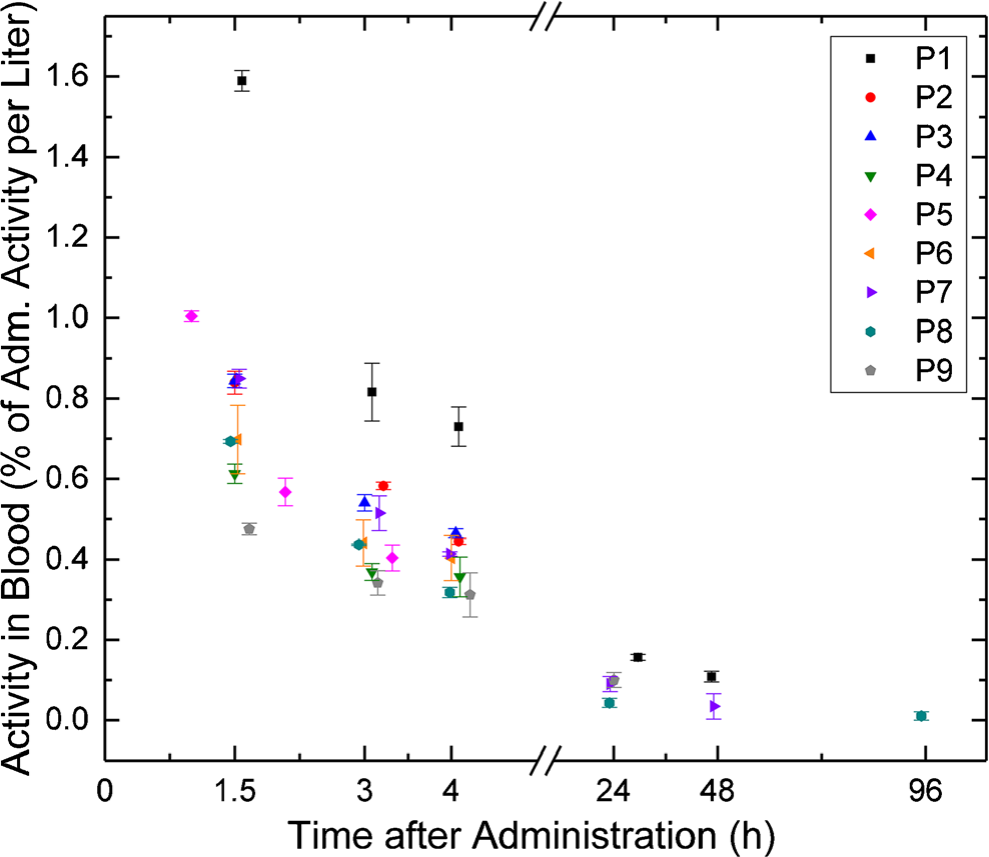
Activity in the blood as a function of the time after administration

### Activity quantification

Up to 3 ml of each radioactive blood sample was measured in a calibrated, high-purity germanium detector (Canberra, Rüsselsheim, Germany). Several traceable standards with different radionuclides (e.g., ^137^Cs ^152^Eu, ^22^Na, ^131^I, ^177^Lu, ^133^Ba) were used to ascertain the energy and efficiency calibration of the germanium detector in the energy range between 80 keV and 2 MeV. The acquisition duration of each measurement was either 12 h or 24 h. As the acquisition duration is not negligible compared to the half-life of ^223^Ra, an additional correction of the decay during the acquisition to the start time of the measurement was performed. For 12 h and 24 h measurement durations, the correction factors are 1.015 and 1.031, respectively. To quantify the activity of ^223^Ra in the blood, the weighted mean of three γ-emission lines were combined: ^223^Ra at 269.5 keV (emission probability of 14.2%), ^219^Rn at 271.2 keV (emission probability of 11.1%), and ^211^Bi at 351.0 keV (emission probability of 13.0%). By comparing the count rates of the γ-emission lines it was ascertained that there was equilibrium between ^223^Ra and its progeny. The activity value was decay corrected to the corresponding blood sampling time point.

To describe the activity retention in the blood for each patient, monoexponential or biexponential fit functions were used, depending on the number of blood sampling time points.

### Dosimetry

Absorbed doses to the blood *D*_Bl,α_ (*t*) as a function of the time after administration were calculated according to the MIRD formalism [22], considering only the contribution of the self-irradiation of the blood. Furthermore, it was assumed that the energy of all α-particles emitted was deposited locally, while the energy contribution of electrons and γ-radiation was not considered. This resulted in:

$$ {D}_{\mathrm{Bl},\upalpha}(t)={S}_{\upalpha, \mathrm{Bl}\leftarrow \mathrm{Bl}}\cdotp {A}_0\cdotp {\tau}_{\mathrm{Bl}}(t)=15.5\ \mathrm{mGy}\ \mathrm{ml}\ \mathrm{kB}{\mathrm{q}}^{-1}\ {\mathrm{h}}^{-1}\cdotp {A}_0\cdotp {\tau}_{\mathrm{Bl}}(t) $$

*A*_0_ is the administered activity in kilobecquerels and *τ*_Bl_(*t*) denotes the time-integrated activity coefficient (TIAC) for the activity concentration per milliliter blood. *τ*_Bl_(*t*) was obtained for each patient individually by integrating the time-activity function over time from the time of injection up to the time point *t* of the respective blood withdrawal. The absorbed dose rate per unit activity *S*_α,Bl ← Bl_ was taken from [8].

For comparing the patients’ absorbed doses to the blood with the results of a previously performed model-based dosimetry calculation [10], the underlying biokinetic model characterizing the biodistribution of [^223^Ra]RaCl_2_ and its progeny was amended. For this amendment, the number of nuclear transformations was calculated for all α-emitting progeny of [^223^Ra]RaCl_2_ for direct uptake of [^223^Ra]RaCl_2_ into the blood. For this purpose, independent biokinetics of [^223^Ra]RaCl_2_ and its progeny were assumed, taking the different chemical properties of the radionuclides in the decay chain into account, in analogy to the publication by Stephan et al. [23]. The energies of the α-particles per transition were taken from the data published by Schumann et al. [8]. The energy deposition of electrons and γ-radiation was not taken into account due to their low contribution to the total energy imparted (< 5%) [8].

### Immunostaining and analysis of DNA damage tracks

For the evaluation of DNA damage, PBMCs were separated from whole blood by density centrifugation (BD Vacutainer CPT tubes; BD, Heidelberg, Germany), washed, and fixed in 70% ethanol, as described previously [15]. The immunofluorescent staining with γ-H2AX and 53BP1 antibodies and the evaluation of DNA damage was performed as described elsewhere [8]. X-ray irradiated (collected 30 min after 0.5 Gy exposure) lymphoblastoid cells were included as a positive control in all the γ-H2AX+53BP1 immunofluorescent staining procedures to ensure positive assay performance by presence of colocalizing γ-H2AX+53BP1 foci in these cells. The PBMCs analyzed were selected randomly on the cytospin preparations. Only nuclei of cells that were morphologically intact and did not to overlap with other cells were analyzed.

Besides distinct small foci, γ-H2AX+53BP1-positive tracks and large foci (Ø > 1.1 μm) were observed in the cell nuclei. Since the tracks and these large foci only occurred in samples irradiated with α-emitters, the large foci likely resulted from α-hits laying perpendicular to the observed focal plane [8]. The number of the visible tracks and large foci were counted manually in a median of 826 cells (range: 573 cells–930 cells) by the same experienced investigator (H.S.) as “α-tracks”, while small foci were neglected in this analysis.

### Statistical analysis

For statistical analysis and plotting OriginPro 2017 (Origin Lab Corporation, Northampton, MA, USA) was used. Shapiro-Wilk tests were performed to test whether data were normally distributed. As normality was rejected for most data sets, Wilcoxon signed-rank tests were applied to test whether the number of α-tracks was statistically significant at different time points. The standard deviation (SD) of the number of α-tracks per 100 cells was calculated for each sample assuming a Poisson-distribution.

## Results

### Patients and blood sampling

In total, nine patients (P1–P9) aged between 57 and 78 years were enrolled. The administered activity of [^223^Ra]RaCl_2_ ranged between 3.3 MBq and 5.6 MBq, depending on the weight of the patient. In some patients, there were slight deviations from the nominal activity. The demographic and clinical data of the patients, including the blood sampling time points, are listed in Table 1. Regarding the blood sampling, there were occasional deviations from the nominal blood sampling time points, and samples on the days after the treatment (≥ 24 h after administration) could not be taken from all patients.

**Table 1:** Patient demographic and clinical data, including blood sampling time points

Patient ID	Age (years)	Weight (kg)	Administered activity (MBq)	Gleason score	Number of bone metastases	Pretreatments	Nominal blood sampling time points: time after administration
P1	63	95	5.5	9	Disseminated, > 50	Prostatectomy	Before administration, 1.5 h, 3 h, 4 h, 24 h, 48 h
P2	72	103	5.6	8	Approx. 5	None	Before administration, 1.5 h, 3 h, 4 h
P3	76	78	4.6	9	Disseminated, > 50	Prostatectomy, ADT, local RTx,	Before administration, 1.5 h, 3 h, 4 h
P4	78	63	3.3	8	Disseminated, > 50	Prostatectomy, ADT, CTx, RTx	Before administration, 1.5 h, 3 h, 4 h
P5	57	85	4.6	9	Disseminated, > 50	Prostatectomy, ADT, RTx, CTx	Before administration, 1 h, 2 h, 3 h
P6	72	89	4.9	9	Disseminated, > 50	Prostatectomy, ADT, CTx, RTx	Before administration, 1.5 h, 3 h, 4 h
P7	72	90	4.9	9	Disseminated, > 20	Prostatectomy, ADT, RTx	Before administration, 1.5 h, 3 h, 4 h, 24 h, 96 h, 28 d
P8	76	73	4.0	9	Approx. 10	Prostatectomy, ADT, RTx	Before administration, 1.5 h, 3 h, 4 h, 24 h, 48 h, 28 d
P9	70	77	4.2	7	Disseminated, > 200	Prostatectomy, ADT, RTx	Before administration, 1.5 h, 3 h, 4 h, 24 h, 30 d

*ADT*, androgen deprivation therapy; *CTx*, chemotherapy; *RTx*, external-beam radiation therapy

### Activity in the blood

The activity concentration in the radioactive blood samples ranged from 0.4 Bq ml^−1^ (P8, 96 h after administration) to 87.4 Bq ml^−1^ (P1, 1.5 h after administration). The corresponding values are listed in Suppl. Table 1. 4 h after administration, a median of 0.4% of the administered activity (range: 0.3–0.7%) per liter of blood remained. 24 h after administration, the remaining activity per liter blood decreased to a median of 0.1% (range: 0.04–0.16%). The activity concentration in the blood as a function of the time after administration is shown in Fig. 1. For dosimetry calculations, individual monoexponential or biexponential fits were performed. Biexponential fits could only be implemented in patients that provided data for at least two late time points (24 h and 48 h or 96 h after administration), i.e., patients P1, P7, and P8. In these cases, only the physical half-life of ^223^Ra was considered for one component of the biexponential fit function, i.e., physical decay was assumed for later time points.

### Dosimetry

The absorbed doses to the blood up to 4 h after administration were below 6 mGy in all samples. 1.5 h, 3 h, and 4 h after administration, the median of the absorbed dose to the blood was 1.0 mGy (range: 0.5 mGy – 3.2 mGy), 1.8 mGy (range: 0.9 mGy – 4.6 mGy), and 2.1 mGy (range: 1.2 mGy – 5.2 mGy), respectively. The corresponding patient-specific data is listed in Suppl. Table 1. The lack of data for late time points (> 24 h after administration) for some patients impeded an integration of the time-activity curves until *t* = ∞ and therefore the calculation of the total absorbed doses to the blood. Thus, total absorbed doses to the blood were calculated only if the activity retention in the blood could be fitted with a biexponential function, i.e., in patients P1, P7, and P8 that provided data 24 h and 48 h or 96 h after administration. This resulted in a total absorbed dose to the blood of 16.5 mGy (i.e., 3.0 mGy MBq^−1^) for P1, 6.4 mGy (i.e., 1.3 mGy MBq^−1^) for P7, and 4.0 mGy (i.e., 1.0 mGy MBq^−1^) for P8.

By applying the model calculation for the biokinetics of a systemic [^223^Ra]RaCl_2_ application published by Lassmann and Nosske [10], the total numbers of decays per Becquerel resulted in 3159 for ^223^Ra, 3694 for ^219^Rn, 3694 for ^215^Po, 4022 for ^211^Bi, and 11 for ^211^Po. The resulting total absorbed dose to the blood according to the model calculation was 3.0 mGy MBq^−1^. This value matches the value calculated for P1 in this study.

### DNA damage in PBMCs

**Fig. 2:**
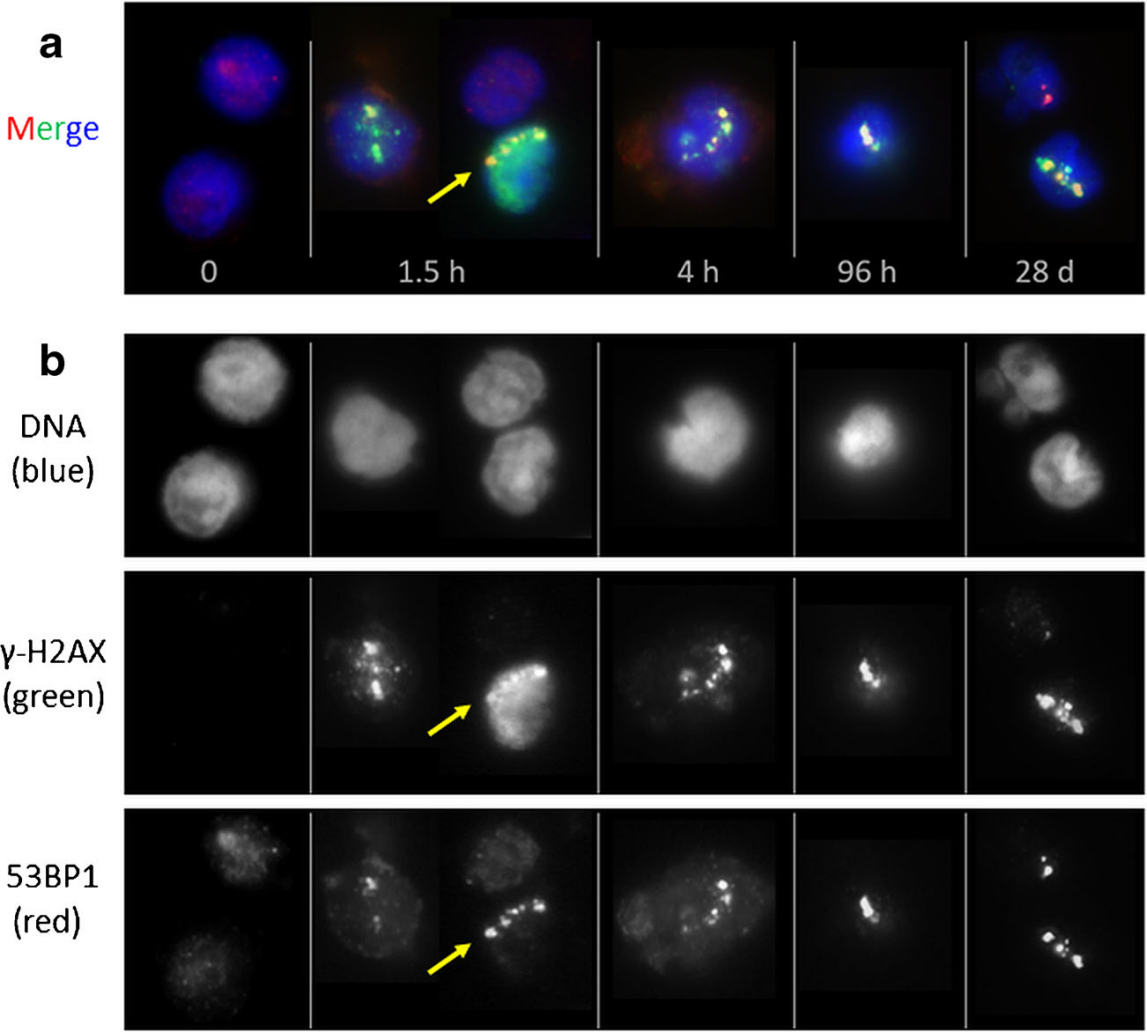
**a** Selection of PBMC nuclei (DNA stained with DAPI, blue) with α-particle hits (α-tracks) stained for γ-H2AX (green) and 53BP1 (red), collected from different patient samples at successive times points after [^223^Ra]RaCl_2_ administration. Cells shown reflect the variable morphologies of DNA damage markers along α-tracks seen among patient samples. Cell nuclei of a baseline sample without α-hits are shown for comparison to the left (0). α-tracks were usually composed of a more-or-less linear succession of large γ-H2AX-positve domains that show partial co-localization with large 53BP1 foci (see [21]). The overall green cell of the 1.5h time point (arrow) displays pan-γ-H2AX staining, typically seen in a fraction of cells after high LET irradiation [24]. The α-particle trajectory in this nucleus can be deduced by the succession of the 53BP1 foci (arrow; see red channel below). **b** Single color channel display of the RGB channel images in **a**, showing the distribution of the individual DSB marker proteins along the α-induced DNA damage tracks. The channel proteins and colors are indicated to the left. The α-track in the pan-γ-H2AX cell is arrowed

α-tracks were observed in all exposed samples at different sampling time points and displayed a variable morphology, with γ-H2AX usually marking the damaged chromatin track or sometimes even the entire nucleus, and 53BP1 forming a succession of large foci or clusters along the γ-H2AX-marked presumed particle track (Fig. 2), which agrees with previous reports [21, 24]. The damage patterns observed along the α-tracks in PBMC nuclei of different samples and of different patients were of variable morphology. Figure 2 shows a selection of PMBC nuclei showing typical signal patterns of γ-H2AX and 53BP1 along α-tracks in PBMC nuclei of different patients.

In the baseline samples, which were taken before administration, γ-H2AX and 53BP1 staining revealed in average four α-tracks in 7382 counted cells of all baseline samples (average frequency of 0.05%). Six control samples did not display α-tracks.

**Fig. 3:**
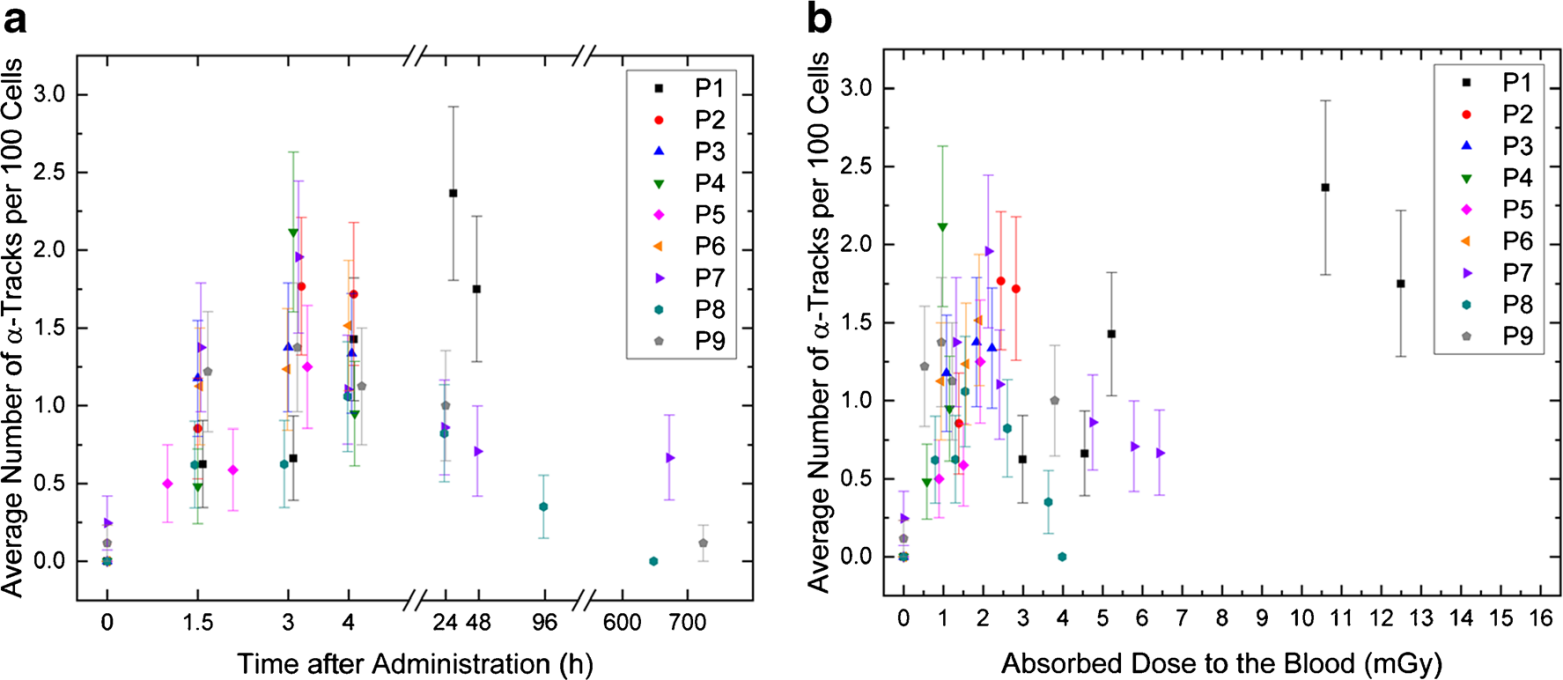
**a** Average number of α-tracks per 100 cells as a function of the time after administration. **b** Average number of α-tracks per 100 cells as a function of the absorbed dose to the blood

PBMCs of patients after ^223^Ra administration were found to display γ-H2AX and 53BP1-positive α-tracks (Fig. 2), with the average number of α-tracks per 100 cells in the irradiated samples ranging from 0 to 2.4. Usually, there was only one α-track per cell hit. At 1.5 h, 3 h, and 4 h after administration, the mean of the average number of α-tracks per 100 cells was 0.93 ± 0.33, 1.37 ± 0.52, and 1.28 ± 0.26, respectively. Compared to baseline, the average α-track frequency was significantly increased in the samples taken 1.5 h (*p* = 0.008), 3 h (*p* = 0.004), and 4 h (*p* = 0.008) after administration. A significant increase of the α-track frequency was also observed between 1.5 and 3 h after administration (*p* = 0.008). 24 h after administration, the average number of α-tracks per 100 cells ranged between 0.82 (P8) and 2.37 (P1). Four weeks after administration, the average number of α-tracks per 100 cells was 0.67 in P7 and 0.12 in P9, while no α-tracks were detected in the 4 weeks sample of P8. The average number of α-tracks per 100 cells is listed in Suppl. Table 1 for each patient individually. The average number of α-tracks as a function of the time after administration is shown in Fig. 3a. An additional overview is given in Suppl. Fig. 1.

**Fig. 4:**
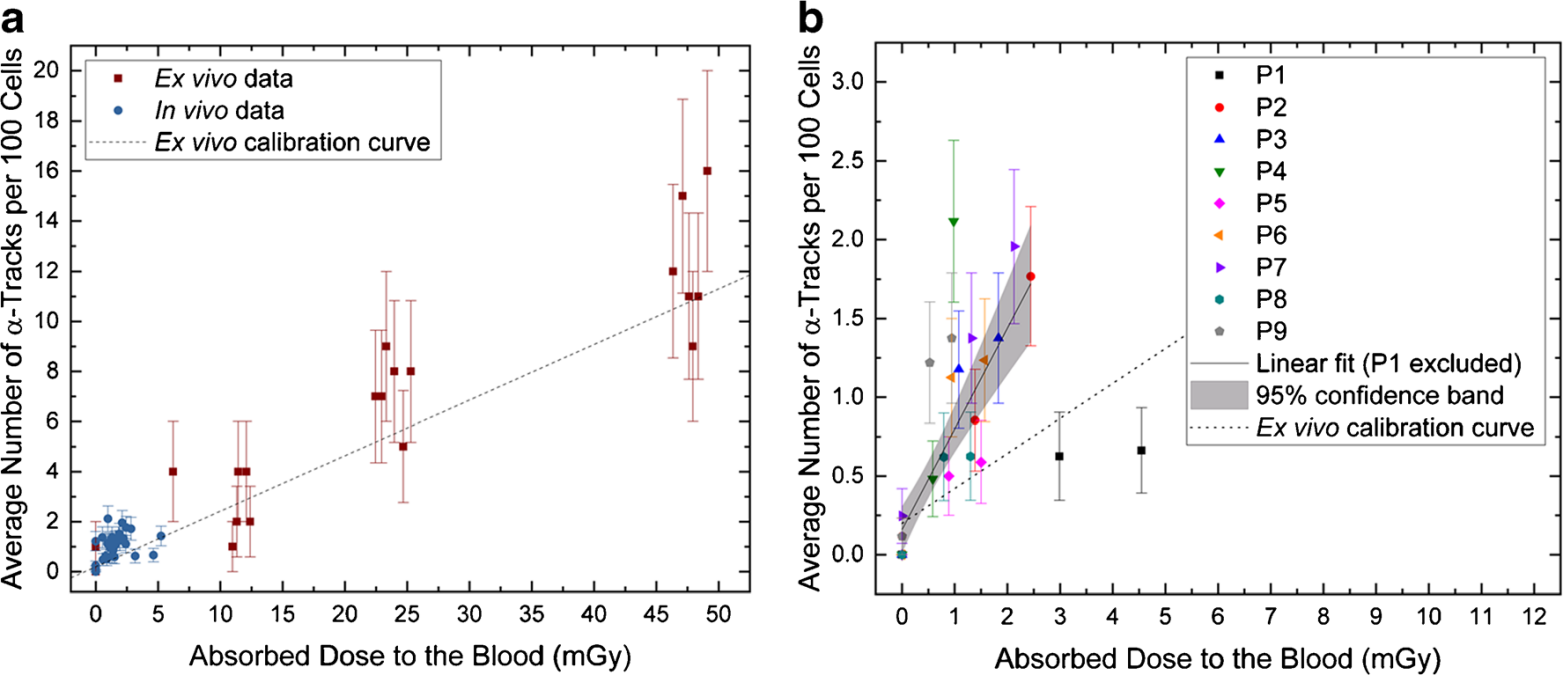
Average number of α-tracks per 100 cells as a function of the absorbed dose to the blood in the first hours after administration. **a** Comparison between the pooled in vivo data of the nine patients (blue; data points up to 4 h after administration are shown) and ex vivo data collected in a previous study (red; only the data points up to an absorbed dose to the blood of 50 mGy are shown) [8]. The number of α-tracks in vivo is higher than predicted by the straight line obtained by the ex vivo calibration (dotted line). **b** Individual patient data up to 3 h after administration with a linear fit (solid line) to the pooled data, including a 95% confidence band (gray area). The data of patient P1 was excluded. The ex vivo calibration curve [8], (dotted line) is shown for comparison

In Fig. 3b, the average number of α-tracks as a function of the absorbed dose to the blood is plotted. Based on the results of previous studies [14, 16, 17], a dose-dependent increase in α-tracks in the first hours after therapy start was expected. A comparison with previously published ex vivo data [8] is given in Fig. 4a. The ex vivo irradiation was accomplished by adding [^223^Ra]RaCl_2_ solution with different activity concentrations to blood samples taken from volunteers and irradiating the blood ex vivo for 1 h at 37 °C on a roller mixer. The evaluation of the ex vivo α-track DNA damage revealed that the relationship between the number of α-tracks and the absorbed dose to the blood in the range between 6 mGy and 136 mGy is described, with sufficient accuracy, by a linear relationship [8]. The corresponding ex vivo calibration curve and the data points up to an absorbed dose to the blood of 50 mGy are plotted in Fig. 4a. Compared to the ex vivo linear calibration curve, the average frequency of α-tracks in the dose range < 3 mGy in vivo was higher than expected, while the data of P1, who showed absorbed doses to the blood of  ≥ 3 mGy to the blood at all time points after administration, were more consistent with the ex vivo results [8]. In order to implement an in vivo calibration curve, as established in previous studies with β-particle emitting radionuclides [14, 16–18], a linear fit was performed (Fig. 4b). For this fit, the pooled patient data up to 3 h after administration in the dose range < 3 mGy were considered, i.e., the data of P1 were excluded from the analysis. This resulted in the following linear equation (*R*^2^ = 0.71):

$$ \mathrm{Average}\ \mathrm{number}\ \mathrm{of}\ \upalpha -\mathrm{tracks}\ \mathrm{per}\ 100\ \mathrm{cells}=\left(0.159\pm 0.072\right)+\left(0.640\pm 0.088\right)\ \mathrm{mG}{\mathrm{y}}^{-1}\cdotp {D}_{\mathrm{Bl},\upalpha} $$

Compared to the slope of the linear ex vivo calibration curve [8], this slope is 2.9 times higher.

## Discussion

Here we present, to our knowledge, the first investigation of the in vivo induction of DNA double-strand break damage tracks in PBMCs of patients during therapy with the α-particle emitting radiopharmaceutical [^223^Ra]RaCl_2_. ^223^Ra treatment of castration-resistant prostate cancer patients has been observed to prolong lives of patients [4, 5]. It can lead to prolonged internal irradiation of bone, blood, and other organs [10, 11], which may induce hematotoxicity [11, 25]. The bone-seeking properties of ^223^Ra lead to exposure of the bone marrow and the exposure to α-emitters can lead to DNA damage [8, 9] and complex chromosome aberrations [6, 7].

Sakane et al. [26] previously reported the consistency between the results of two different markers for radiation damage, γ-H2AX, and chromosomal aberrations (CAs) for low-dose chest CT, thus linking results of the two assays. Furthermore, a link between CA frequency and cancer risk was established in an epidemiological study by Bonassi et al. [27]. Both studies, therefore, imply a potential link between DNA double-strand breaks and long-term cancer risk; however, as Brenner pointed out in a recent editorial, that considering all the available evidence to date, such a causal link is still not well established [28].

In blood samples taken after the administration of [^223^Ra]RaCl_2_, PBMC nuclei with DNA damage tracks were detected that often displayed a variable morphology, with γ-H2AX being the most reliable marker of the damaged chromatin track. Both γ-H2AX and 53BP1 often formed a succession of large (super) foci along the presumed particle track, a feature that may relate to distribution of different chromatin moieties (eu-, heterochromatin) in the nucleus, chromatin motions [19, 29–31], and/or to preparation variables like antibody binding and chromatin compaction [21, 32–34].

We not only obtained results for the time-dependency of DNA damage induction but also investigated the dependency on the absorbed dose to the blood. The absorbed doses to the blood calculated in this study were in the very low dose range, with a maximum total absorbed dose to the blood of 16.5 mGy. The absorbed doses to the blood up to 4 h after the administration of the radiopharmaceutical were even below 5 mGy. However, their exact calculation is limited by the low number of blood sampling time points as well as by the rather low activity concentrations in the blood. Regarding the activity concentrations in the blood, we observed a high inter-individual variation between the patients included in this study, implying large differences in absorbed dose values. Overall, the clearance of [^223^Ra]RaCl_2_ from the blood was rapid, which is in good agreement with the results of Carrasquillo et al. who investigated the pharmacokinetic and biodistribution of [^223^Ra]RaCl_2_ in ten patients [11]. They observed that 4 h after administration a median of 2.0% of the administered [^223^Ra]RaCl_2_ activity was still in the patients’ plasma. 24 h after administration, it had decreased to a median of 0.55%. Assuming a plasma volume of 3.2 l, as stated in their publication as the median plasma volume, and the given plasma to blood activity concentration ratio of 1.5, this results in 0.4% and 0.1% of the administered activity per liter blood remaining in the circulation 4 h after administration and 24 h after administration, respectively [11]. These values are in accordance with the values obtained in our study, which emphasizes the reliability of the activity concentrations determined in our study that were used for the absorbed dose calculations.

Our γ-H2AX+53BP1 immunofluorescence data show an increased frequency of α-particle-induced DNA damage tracks in blood PBMCs after administration of [^223^Ra]RaCl_2_. It appears that the γ-H2AX and 53BP1 assay is suitable to detect DNA damage even after α-exposures in the very low dose range. The in vivo specificity of the track DNA damage geometry for PBMC nuclei hit by α-particles renders the γ-H2AX assay a potent method for the verification of incorporation of α-emitting radionuclides or radionuclide mixtures in radiation accidents or malevolent events.

We are aware that our study is somewhat limited by the low number of included patients (*n* = 9), caused by recruitment problems and individualized treatment schemes. Due to the low number of patients, it was not possible to investigate a correlation between DNA damage in blood cells or the absorbed doses to the blood and the disease burden or pretreatment of the patients in this study. Furthermore, since the very low absorbed dose to the blood entails a very low frequency of α-track events, this low number of events may lead to relatively large statistical uncertainties. To reduce the uncertainties of the enumeration in the very low dose range below 3 mGy, we increased the number of counted cells to 573–930 cells per sample, relative to the 100 cells per sample in our previous ex vivo studies that focused on higher doses [8, 9].

The number of α-tracks in this very low dose range was higher than expected based on the ex vivo calibration curve of our previous study [8], as shown in Fig. 4, with the slope of the current in vivo calibration curve being 2.9 times higher than the slope of the ex vivo calibration curve [8]. This result may indicate a low dose hypersensitivity, as suggested in previous studies [18, 35, 36]. However, we cannot exclude that our results are somewhat affected by methodological limitations as discussed above. Furthermore, it should be noted that a direct comparison to the ex vivo data is difficult as the latter comprised a dose range from 6 mGy to 136 mGy, while the behavior in the very low dose range remained unexplored.

So far, there are only few studies reporting on radiobiological effects of [^223^Ra]RaCl_2_ [37, 38]. Due to different irradiation set-ups, cell types, and dose ranges, however, a direct comparison to our study is not possible. Runge et al. [39] investigated the repair proficiency in lymphocytes of prostate cancer patients over six therapy cycles with [^223^Ra]RaCl_2_ by quantifying γ-H2AX foci before each cycle, but did not analyze the induction of the DSBs during the therapy. Contrary to our study, they did not observe any α-tracks 4 weeks after the treatment. In our study, the low but still elevated numbers of α-tracks in most of the late samples (24 h up to 4 weeks after administration) possibly indicate incomplete or arrested DNA repair in some circulating PBMCs, since α-induced DNA damage is considered to be complex and difficult to repair [40, 41]. In this respect, it has been realized that α-induced DNA damage poses a nearly insolvable task for the DNA repair machineries of the cell [41, 42], which could lead to the persistence of DNA damage tracks in long-lived PBMCs. Further investigations on the DNA repair factors and pathways active along α-tracks in nuclear DNA may lead to better understanding of repair processes active in α-damaged chromatin as indicated by recent super-resolution light microscopy [21, 43] or transmission electron microscopy studies [44, 45].

Up to now, there is only one investigation studying the biological effects in PMBCs during therapy with α-emitters. In this study, Stephan et al. determined the frequency of chromosomal aberrations and the absorbed dose to the blood in ankylosing spondylitis patients who were undergoing treatment with [^224^Ra]RaCl_2_ with a total administered activity of 10 MBq (10 i.v. injections of 1 MBq per week) [23]. For this treatment, the absorbed dose coefficient to the blood was 4.7 mGy MBq^−1^ [23] compared to 3.0 mGy MBq^−1^ according to the model calculation for ^223^Ra in the current study. With respect to chromosomal aberrations, Stephan et al. could show an absorbed dose-dependency of the number of dicentrics in PMBCs during therapy, increasing with almost each 1 MBq injection of [^224^Ra]RaCl_2_. As our study was limited to the first hours in the first therapy cycle and the respective change of α-tracks over time and absorbed dose after one injection of about 5 MBq [^223^Ra]RaCl_2_, a direct comparison of the biological effects is not possible, also because of the different endpoints studied. However, since our two ex vivo studies showed that the induction of α-tracks is identical for both radium isotopes [8, 9], we would expect similar α-track induction and decay patterns for both isotopes in vivo.

## Conclusion

This in vivo study on [^223^Ra]RaCl_2_-induced DNA damage shows that α-tracks appearing in PBMCs can be detected at very low absorbed doses to the blood, highlighting the high sensitivity of the γ-H2AX+53BP1 DNA damage assay after internal exposure. Even at absorbed doses to the blood of less than 16 mGy and even below 3 mGy, there was an increased frequency of PBMCs with α-particle-induced DNA damage, which correlated with the absorbed dose to the blood in the first hours after administration. In some cases, α-tracks were detectable even 4 weeks after the treatment. However, further studies are needed to investigate the effects of incomplete DNA damage repair after multiple treatment cycles. Overall, this assay may prove useful in accident scenarios or for the estimation of DNA damage during radionuclide therapy, also with other α-emitting radiopharmaceuticals.

## Supplementary information

ESM 1 (PDF 241 kb)

## Data Availability

The data sets generated and analyzed in the course of the current study are available from the corresponding author on reasonable request.
