# Supplementary material for: α-Particle-induced DNA damage tracks in peripheral blood mononuclear cells of [223Ra]RaCl2-treated prostate cancer patients
Source: Eur J Nucl Med Mol Imaging. 2021 Feb 4;48(9):2761–70. doi: 10.1007/s00259-020-05170-6 (PMC8263441; doi:10.1007/s00259-020-05170-6)
Supplement: Supplementary file 1 — (PDF 241 kb) [file 259_2020_5170_MOESM1_ESM.pdf]

α-particle-induced DNA damage tracks in peripheral blood mononuclear cells of [^{223}Ra]$RaCl_2$-treated prostate cancer patients – Supplementary Material

S. Schumann[1]*, U. Eberlein[1]*, C. Lapa[1,2], J. Müller[3], S. Serfling[1], M. Lassmann[1†], H. Scherthan[3†]

1) Department of Nuclear Medicine, University of Würzburg, Würzburg, Germany
2) Nuclear Medicine, Medical Faculty, University of Augsburg, Augsburg, Germany
3) Bundeswehr Institute of Radiobiology affiliated to the University of Ulm, Munich, Germany

* Shared first authorship

† Shared senior authorship

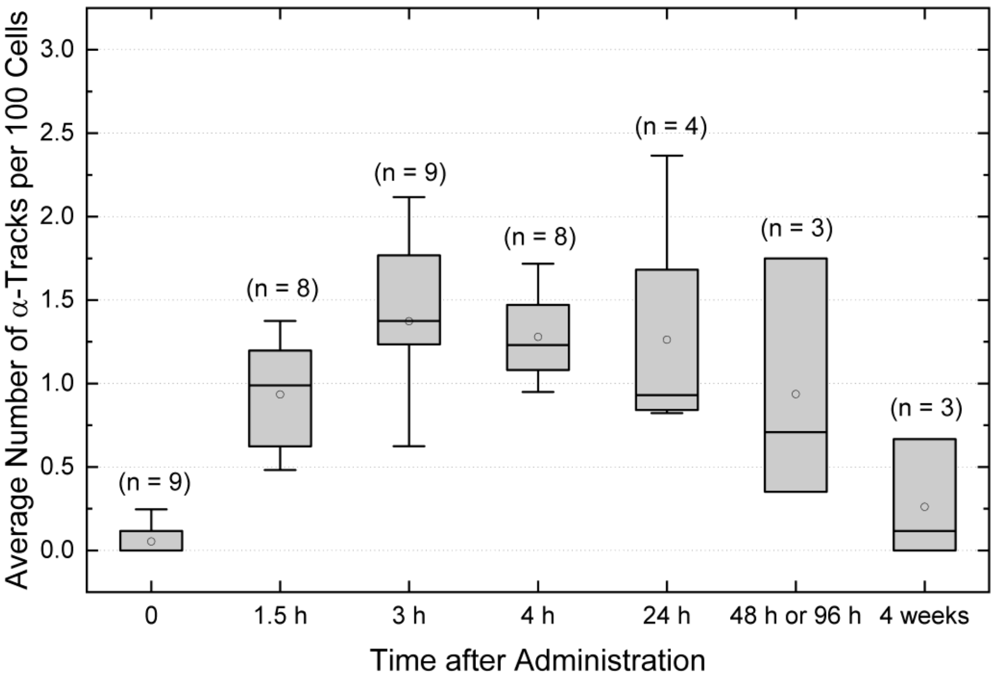

Suppl. Figure 1: Boxplot of the average number of α-tracks per 100 cells at the different blood sampling time points. A significant increase of the average α-track frequency was observed in the samples taken 1.5 h ($p=0.008$), 3 h ($p = 0.004$) and 4 h ($p = 0.008$) after administration relative to baseline (0) and between 1.5 h and 3 h after administration ($p = 0.008$).

Suppl. Table 1: Blood sampling time points, results of the activity measurements, the absorbed dose calculations and the enumeration of α-tracks.

Patient	Exact blood sampling time point: time after administration (h)	Activity concentration in the blood (Bq ml^{-1})	Absorbed dose to the blood up to this time point (mGy)	Average number of α-tracks per 100 cells	SD of the average number of α-tracks per 100 cells
P1	before administration	0.0	0.0	0.00	0.00
	1.6	87.4	3.0	0.63	0.28
	3.1	44.9	4.5	0.66	0.27
	4.1	40.2	5.2	1.43	0.40
	29.6	8.6	10.6	2.37	0.56
	46.6	6.0	12.5	1.75	0.47
P2	before administration	0.0	0.0	0.12	0.12
	1.50	46.3	1.4	0.85	0.32
	3.22	32.1	2.4	1.77	0.44
	4.08	24.5	2.8	1.72	0.46
P3	before administration	0.0	0.0	0.00	0.00
	1.50	38.3	1.1	1.18	0.37
	3.00	24.5	1.8	1.38	0.41
	4.05	21.1	2.2	1.34	0.39
P4	before administration	0.0	0.0	0.00	0.00
	1.50	20.0	0.6	0.48	0.24
	3.08	12.0	1.0	2.12	0.51
	4.10	11.6	1.2	0.95	0.34
P5	before administration	0.0	0.0	0.00	0.00
	1.00	45.2	0.9	0.50	0.25
	2.08	25.5	1.5	0.59	0.26
	3.32	18.2	1.9	1.25	0.40
P6	before administration	0.0	0.0	0.00	0.00
	1.53	33.6	0.9	1.13	0.38
	2.98	21.3	1.6	1.23	0.39
	4.00	19.4	1.9	1.52	0.42
P7	before administration	0.0	0.0	0.25	0.17
	1.6	41.3	1.3	1.38	0.41
	3.2	25.0	2.1	1.96	0.49
	4.0	20.1	2.4	1.10	0.35
	23.1	4.4	4.7	0.86	0.30
	47.0	1.7	5.8	0.71	0.29
	671.8	-*	6.4	0.67	0.27
P8	before administration	0.0	0.0	0.00	0.00
	1.5	27.7	0.8	0.62	0.28
	2.9	17.4	1.3	0.63	0.28

	4.0	12.7	1.5	1.06	0.35
	23.0	1.7	2.6	0.82	0.31
	95.0	0.4	3.6	0.35	0.20
	648.1	-*	4.0	0.00	0.00
P9	before administration	0.0	0.0	0.12	0.12
	1.7	19.9	0.5	1.22	0.39
	3.1	14.3	0.9	1.38	0.41
	4.2	13.1	1.2	1.13	0.38
	24.0	4.2	3.8	1.00	0.35
	723.9	-*	-**	0.12	0.12

* Activity values for the time points 4 weeks after administration were not measured.

** The total absorbed dose to the blood was not calculated due to the missing data at the time point 48 h or 96 h after administration.
